# Understanding the impact of demand shocks on the container port industry

**DOI:** 10.1057/s41278-022-00222-0

**Published:** 2022-03-07

**Authors:** Daniele Crotti, Claudio Ferrari, Alessio Tei

**Affiliations:** 1 Department of Economics, University of Insubria, Varese, Italy; 2 Department of Economics, University of Genova, Via Vivaldi 5, 16126 Genova, Italy

**Keywords:** Liner shipping, Competition assessment, Integration, Non-cooperative games

## Abstract

The Covid-19 pandemic has severely impacted the world economy, generating an unprecedented shock that pushed carriers to adapt to the collapse of demand. Most of the related adaptation actions (e.g., blank sailings) appear as temporary initiatives being insufficient to reach a long run equilibrium. Moreover, while carriers managed to adjust their own supplied capacity to the ongoing crisis, the port sector has been greatly impacted by the fall in transport demand, not being able to counteract the demand shortages as effectively as the carriers. Against this backdrop, the paper introduces a model for assessing the effects of demand shocks (e.g., due to the pandemic) on the integration strategies of carriers. We focus on the possible initiatives that demand shocks may trigger on the horizontal and vertical integration among the actors of the shipping industry. In doing so, the present study provides useful insights for better understanding potential future market modifications and their impact on social welfare. Using non-cooperative games, profit-maximising strategies, in case of such integrations, are compared in order to study how carriers and terminal operators might react to demand shocks in the medium and long run.

## Introduction

The spread of the Covid-19 virus has deeply impacted several industries and economies, causing modifications in global value chains and in several trade-related sectors.

The effect of lockdowns on regional economies was also amplified by the different starting and ending dates of local restrictions: several Asian countries entered in first partial lockdowns in February 2020, most European countries imposed lockdowns starting in mid-March of the same year, while the American countries implemented first restrictions about 2 weeks later.

This sudden change of production and distribution patterns impacted the economy severely. IMF (2021) estimated the global economy to shrink by 3.3% in 2020, with a rebound in 2021 to be evidenced by the re-opening of most activities. Similarly, UNCTAD (2020) estimated declines in the maritime industry of about 4.1% in 2020.

The effect of shutting down production and shops of non-essential goods had an important impact on transport and logistics systems. In our view, the impact of the pandemic on trade and maritime business has demonstrated once more how a better understanding of the effect of external shocks on the shipping market could improve the responses of different market players. As such, the Covid-19-related economic lockdowns presented us with a demand shock that we will use here to improve our understanding of strategic behaviour of carriers.

Shipping companies continued to operate even during the most severe phases of national lockdowns, but inevitably the industry was affected by the contraction in production and reduced demand for raw materials and finished goods. Existing literature highlights the difficulties of carriers to adapt to network disruptions and market shocks (e.g., Chen et al. 2016; Achurra-Gonzalez et al. 2019). This implies that demand shocks, such as pandemics have potential long-term consequences. Such conclusions are highlighted by most of the preliminary studies, published in late 2020 on the impact of Covid-19, on either the general shipping business (e.g., Notteboom et al. 2020) or on specific market segments (e.g., Rahman et al. 2020), which hint at these potential long-term impacts. Notteboom and Haralambides (2020) point out that the pandemic substantially changed the competitive playing field for the port sector, and increased uncertainty for ports with regard to planning and investments. Ferrari and Tei (2020) argued that the shipping lines were capable of adapting to the Covid-19 pandemic better than in other demand-related crises (e.g., 2009 financial crisis), using a series of novel and partially collaborative approaches (e.g., massive use of the so-called blank sailings, i.e., the possibility to cancel a port call or even a service, as a solution for adapting to demand fluctuations).

As stated by Wilmsmeier and Sánchez (2015), economic downturns strongly affect the shipping lines’ strategies, especially in terms of network hierarchization. In the aftermath of economic and financial crises, the shipping and port industries have often reacted through a series of market adaptation strategies. Related to this, several studies (e.g., Midoro and Pitto 2000) for shipping and Slack and Fremont (2005) for ports) define horizontal and vertical integration as a key corporate strategy to rationalise costs and expand the companies’ market share. Alexandrou et al. (2014) reviewed a series of mergers & acquisitions (M&As) in the shipping industry, to determine their effects on company performance, depending on local regulation and conditions. As such, integration plays a key role in the value generation of most shipping companies and in the possibility for them to compete in the market. This has been highlighted by relevant regulatory bodies, as in the case of strategic partnerships (e.g., alliances). For instance, the European Commission granted the shipping sector exemptions from “normal” competition regulation, often justified on the basis of the cost structure peculiarities (OECD 2015). Within this framework, the reaction of most carriers to the Covid-19 pandemic has been developed through alliance-related strategies (e.g., blank sailings and re-routings), underlining in this way how integration is not only a potential solution in business-as-usual situations but it can also play a crucial role in mitigating demand shocks as well (e.g., Ferrari and Tei 2020). These elements urge scholars to understand better the impact of such strategic moves on the economic welfare of the port regions.

Furthermore, on the one hand the role of the integration approaches as a potential way for adapting and recovering from external shocks is relatively understudied; on the other hand, the market impact of such strategic moves, in terms of (port) economic welfare and not just companies’ revenues, is missing. As such, the current paper tries to fill this gap, applying a non-cooperative game to a market-related case study. Thus, the outcome of the proposed analysis will shed lights on the impact on economic surplus of different strategic moves, usually introduced by major market players within the port and shipping competitive environment, to mitigate demand shock effects.

The reminder of paper is as follows. After this brief introduction, Sect. 2 introduces the theoretical background, discussing both general competition theory and applications to the maritime sector. Section 3 is focused on the model development and analysis and the paper concludes with a final section summing up findings and critical discussing the main research outcomes.

## Theoretical background

### Integration and market concentration

Looking at vertical integration strategies, the economic literature often highlights that, when upstream and downstream companies are integrated through governance and/or ownership links, mark-ups or double marginalisation (i.e., the needed profit margin applied by the various companies involved within a specific supply chain) are removed, thus increasing total profits and consumer welfare (Hamilton and Mqasqas 1996; Spengler 1950). On the one hand, vertical integration can help integrated firms to gain larger profits but also to exert more bargaining power (Riordan 2008; Rey and Tirole 2007). On the other hand, this result might not hold when the downstream competition is fierce (Mathewson and Winter 1984) or if vertical integration enhances efficiencies along the market chain, whereas downstream strategies induce lower prices (Chen 2001). Recently, Slade and Kwoka Jr (2020) argued that vertical integration is not always necessary to achieve the benefits of the elimination of the double marginalization problem, unless vertical mergers induce merger-specific cost savings. Within the shipping sector, multiple reports (e.g., van de Voorde and Vanelslander 2009; ITF 2018; Hoffmann and Hoffmann 2021) have discussed the impact of both horizontal and vertical integration in terms of changing market power as well as the impact in terms of service quality, deployed capacity, and overall trade costs.

Contrasting results about vertical relationships also emerged when considering the competitive effect of *partial* ownerships, that is, in case of upstream (downstream) companies which own and control (or partially own and control) shares of downstream (upstream) firms, conducing to forward (backward) vertical ownership (Brito et al. 2016). Furthermore, more refined analyses distinguish between active (or control) and passive (or silent) ownerships, where the main difference is that, in case of *active* shares, the partially acquired companies might look after the interests of the acquiring ones by internalizing their own gross profits (including vertical-related revenues) in proportion to the detained shareholdings (O’Brien and Salop 2000). Focusing on passive and backward ownership only, Greenlee and Raskovich (2006) confirmed previous research (e.g., Fronmueller and Reed 1996) arguing that silent interests held by a downstream firm yield a partial rebate of the upstream margin, so that input demand increases with backward ownership, and the upstream firm optimally responds by raising prices.

Moving to forward vertical links where companies exert forms of control over partially integrated firms, Baumol and Ordover (1994) showed that when an upstream firm does control, but only partially owns, a downstream firm, then final prices may rise. Essentially, a large part of the literature converges to the conclusion that passive ownerships (where integrated companies are only interested in additional revenues coming from shareholdings) might exacerbate market power issues and do not affect positively consumer welfare. With respect to backward and controlling vertical integration—that is, the primary literature our analysis contributes– the results obtained in the literature display opposite directions, depending on the type of competition. Riordan (1998) showed that, in Bertrand markets, backward vertical integration by a dominant firm raises the competitive fringe's cost and always harms consumers through higher prices However, extending Riordan's analysis to Cournot competition, Loertscher and Reisinger (2014) found that vertical integration is more likely to benefit consumers, i.e., by enlarging the supplied output, when the industry is more concentrated. Similar results were recently derived by Brito et al. (2016), who studied an industry where downstream firms partially own a supplier. Focusing on consumer surplus under passive ownership, the final consumer’s welfare is invariant with respect to ownership shares. By contrast, if ownership comes with partial and active control (as defined in O’Brien and Salop 2000), then consumer surplus is higher and increasing function of the shares. Interestingly, those results are based on two effects of backward partial ownerships that are going to be referred to in our analysis. On the one hand, this entails a vertical-control effect, which reduces the extent of double marginalization, and can enhance both the total output and the consumer welfare. On the other hand, a *tunnelling effect* is also at play, whereby downstream firms use wholesale prices to transfer value from independent and passive upstream shareholders. In other words, the conclusive impact of the partial acquisition of shares happens to be closely related to the qualification of shareholders, where downstream operators controlling upstream firms may erode surplus from the non-controlling ones. Actually, those two contrasting effects are key ingredients of our analysis, where: (i) upstream firms’ shares owned by downstream operators are given, and (ii) the strategic linkage between integrated companies is proportional to shareholdings. Yet, since we are interested in looking at the impact of controlling shares on total throughput and on incentives for horizontal mergers in the liner shipping industry, the second contribution of this paper is devoted to the vertical and horizontal integrations in the maritime sector, as a persistent process which has taken place in the last years (e.g., Crotti et al. 2020; Zheng and Luo 2021).

### An application to shipping

Strategic alliances—and related integration strategies—have achieved unprecedented importance, with nearly 80% of the global container capacity offered by the three main alliances (ITF 2018), up from the roughly 40% in the early ‘00 s. Moreover, only few major maritime routes still see independent carriers offering more than 5% of the deployed capacity (ITF 2019) with some regional markets (e.g., Europe) that are dominated by global players as well. Strategic alliances often impact also the operability of main terminals, with main carriers (e.g., Maersk Line, MSC, CMA-CGM, Cosco) directly or indirectly owning shares of major container terminals.

Regarding to the emergence of vertical cooperation along the maritime supply chain, various scholars have discussed the different levels of integration of carriers with key ports and local/global terminal operators to achieve different types of efficiency at container terminals. Similar to shipping lines, terminal operators and port authorities are also trying to integrate with different industry partners (Rodrigue and Notteboom 2009; Notteboom 2002). As for the ability of removing double marginalization effects through backward integration, Midoro et al. (2005) argued that investing in ports can help liner companies to meet shippers’ needs for efficiency, together with higher reliability, freight control, and even risks (Notteboom and Rodrigue 2012). Among others, Ferrari et al. (2008) showed that servicing home markets affects vessel deployment over trade lanes, enhancing vertical links between terminal operators in Europe and Asia. In the last years, a key decision of liner companies has been whether to manage dedicated terminals and keep them exclusive, or to have a dedicated terminal accessible to all the carriers. Haralambides et al. (2002) found that carriers with dedicated terminals would benefit from such arrangements, as they could enjoy greater flexibility, reliability, short turnaround times, and enhanced efficiency in the management of global supply chains. More recently, Kaselimi et al. (2011) discussed the influences of dedicated terminals on the efficiency of port operations from the perspective of terminal operators and port authorities, using spatial competition models.

Terminal operators can increase their revenues and capacities by offering dedicated terminals, while shipping lines that do not have their own dedicated terminals, generally pay higher fees in non-dedicated terminals. Complementing that analysis, Álvarez-Sanjaime et al. (2013) studied the motives of vertically integrated carriers to extend terminal services to rival shipping lines, and categorized different ways of using carriers’ own terminals. They found that carriers should operate their own terminals in a non-exclusive way, to pursue higher profits. Actually, carriers not owning terminals may also be better off with non-exclusive terminals, resulting in higher port fees, social welfare and industry profits. From a port governance perspective, Yip et al. (2014) developed a game model to study the effects of competition for seaport terminal awards, concluding that terminal operators would prefer to control more terminals in a certain region, and expand their operations to every port. Also, when a port authority has significant market power, the authors argued, it tends to introduce inter- and intra-port competition. Recently, Zhu et al. (2019) emphasized the important role of vertical integration between carriers and terminals to achieve larger synergies in the maritime industry. They developed an analytical model to study the effects of vertical integration, with a focus on shipping lines’ investments in port capacity. They concluded that vertical integration between terminal operators and shipping lines leads to higher port capacity, market output and consumer surplus.

Although the above literature has often found that backward integration between carriers and terminal operators could improve the overall port sector, increasing profits and social welfare, research has not been devoted to evaluating existing incentives for mergers between liner shipping companies in the presence of vertical integration, although the topic has been debated in the modern maritime industry (e.g., Meersman et al. 2015). In a relatively early work, Alix et al. (1999) argue that mergers are effective and relatively immediate strategies to reacting to demand shocks. Similarly, Das (2011) found that, with respect to strategic alliances, mostly during market recessions, carriers are more likely to opt for mergers. Aiming at exerting insights about the impact of merger waves on the stability of alliances, Crotti et al. (2020) found that, for increasing vertical integration between carriers and terminal operators, horizontal integrations among carriers might strengthen the stability of strategic alliances for many levels of market demand, resulting in welfare-enhancing outcomes in the industry. Thus, assessing whether gains from horizontal integration could be affected by demand conditions (or shocks) is of utmost importance among port players in the light of the effects of the Covid-19 pandemic.

## Model and results

By leaving aside the occurrence of strategic alliances and focusing on the effects of both demand conditions and vertical ownerships (in this case, backward) on the incentives to merge, we focus on two port-related scenarios, one in which there is only one terminal owned by a shipping company,^1^ and the other in which there are two competing terminals: a vertically integrated one and a pure stevedoring terminal, and where competition is based on price.^2^ In order to represent the impact of Covid-19 on companies’ behaviour, our main hypothesis is that either demand variations (shocks) or modifications of the shares owned by the integrated companies would impact on the incentive to merge of different carriers, depending on the port configuration. The model outcomes will help in evaluating the impact of different strategies on total port throughput and social welfare, depending on the expected demand shocks. By following a game-theoretic framework, we model horizontal mergers where the carriers, as oligopolistic players in a Cournot competition, might gain efficiencies due to larger capacity (among others, see Perry and Porter 1985; McAfee and Williams 1992; Motta and Vasconcelos 2005; Vasconcelos 2010) and we assume that they (partially or totally) own shareholdings of a private terminal company, in the style of Brito et al. (2016). By discussing the theoretical results of this modelling approach to the best of our knowledge, we aim at providing the sector literature with the first attempt to understand which port setting (between the two presented scenarios) would entail horizontal mergers conducive to higher total port supply and industry surplus as a consequence of demand variations and different shareholding schemes.

This paper investigates the incentives for carriers to merge in port configurations characterized by backward vertical integration between (downstream) carriers and (upstream) terminal operators. To this end, two contrasting scenarios are compared. In the first one, the merging carriers would call at a seaport with one integrated terminal. In the second scenario, there is an integrated and non-integrated terminal, both supplying cargo handling services.

### First scenario with only one vertically integrated terminal

**Fig. 1:**
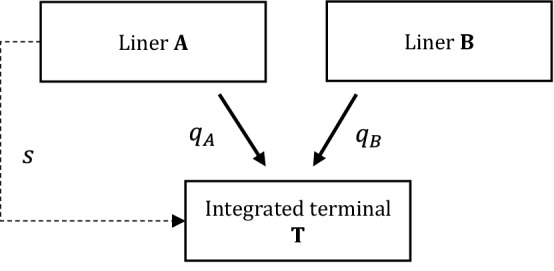
Liner shipping market with an integrated terminal only

In the benchmark market structure, consider two carriers, A and B, supplying homogeneous container shipping services. They are assumed to call at a landlord seaport where a single terminal is operated by an integrated terminal operating company (T) (Fig. 1).

From a shareholding perspective, operator T is assumed to be vertically (backward) integrated with carrier A. In order to have a fairly general model, we assume that A’s shares add up to $s\le 1$, while the residual property rights, $1-s$, are owned by outsider shareholders,^3^ not involved as carriers (e.g., non-integrated terminal operators, investment funds, financial houses, etc.). Since A’s ownership of T has a direct effect on its decisions about output setting (i.e., T is an input supplier for A and B), the vertical relationship is assumed to imply an *active* control by liner A over terminal operations. By contrast, in this model the outsider investors display a *passive* (or silent) ownership, i.e., they only earn additional revenues from T’s business. Clearly, if *s* = 1, A has a total control over T, meaning that A’s overall interests are completely internalized by T. If *s* = 0, instead, all the investors belong to non-shipping sectors. As a result, this approach implies that, when maximizing its objective function, T would consider *active* shareholders’ interests in proportion to their financial stakes in upstream operations (O’Brien and Salop 2000).

Formally, the downstream carriers maximize their own gross profit: ${v}_{A}={\pi }_{A}+s{\pi }_{T}$ and ${v}_{B}={\pi }_{B}$, where ${\pi }_{A}$ and ${\pi }_{B}$ are net (shipping) profits; ${\pi }_{T}$ are T’s profits in port. In turn, as an upstream company, T maximizes the following: ${v}_{T}=s{v}_{A}+(1-s){\pi }_{T}$, i.e., A’s gross profits, weighted for related shares, plus her own profits ${\pi }_{T}$ multiplied by the *passive* $1-s$ shares owned by non-shipping firms (see Brito et al. 2016).

From the shipping market side, for our purposes we model the simplest inverse demand function for shipping services in this scenario, that is: $p=\alpha -Q$, where $\alpha >0$ represents the market size (i.e., demand for container business generated by the specific port), *p* is the level of shipping rates, and $Q={q}_{A}+{q}_{B}$ is the port throughput, calculated as the sum of each carrier’s market output. The carriers compete by setting shipping output (Containers) and bear costs at sea with decreasing returns, i.e., $C\left({q}_{i}\right)={\left({q}_{i}\right)}^{2}/2{k}_{i}$, for $i=A,B$. This quadratic formulation of total costs is general enough to encompass the features of shipping costs (such as congestion and delays due to large outputs; see Zhou et al. 2019) and the capacity endowment ${k}_{i}$, which may convey efficiencies through scale effects. Additionally, carriers must pay terminal fees to T, whose marginal costs are set to zero.

#### Pre-merger game

Before any merger project is envisaged, we derive equilibrium carriers’ outputs and gross profits. The timing of the game is the following. In the first stage, T sets terminal fees (assumed identical for all carriers, regardless of their share in T), while in the second stage, each carrier sets the output to maximize their gross profits. To determine the subgame perfect Nash equilibrium (SPNE) of this two-stage game, we proceed backward, starting from the second stage. For a given level of the terminal fee ${f}_{T}$, and assuming capacity ${k}_{A}={k}_{B}$, normalized to 1, the carriers maximize the following own gross profits:

1 $$\begin{aligned}&{v}_{A}\left({q}_{A},{q}_{B};\,f\right)\triangleq {\pi }_{A}+s{\pi }_{T}=p{q}_{A}-\frac{{\left({q}_{A}\right)}^{2}}{2}-f{q}_{A}+sf\left({q}_{A}+{q}_{B}\right)\\&{v}_{B}\left({q}_{A},{q}_{B};\,f\right)\triangleq {\pi }_{B}=p{q}_{B}-\frac{{\left({q}_{B}\right)}^{2}}{2}-f{q}_{B}.\end{aligned}$$

From (1), carrier A can offset the input (landside) costs and to gain additional revenues from terminal operations (i.e., by increasing $s$, A would reduce the term $-f{q}_{A}$ and raise the term $f{q}_{B}$). Maximizing the above value functions by A and B yields individual and total second-stage equilibrium outputs:

2 $$\begin{aligned} & {q}_{A}(f)=\frac{\alpha }{4}-\frac{f}{4}\left(1-\frac{3s}{2}\right), \quad {q}_{B}(f)=\frac{\alpha }{4}-\frac{f}{4}\left(1+\frac{s}{2}\right)\\ & Q(f) \triangleq {q}_{A}(f)+{q}_{B}(f)=\frac{1}{2}\left(\alpha - f\left(1-\frac{s}{2}\right)\right)\end{aligned}$$

Clearly, higher terminal fees (as wholesale input prices) tend to reduce carriers’ deployed capacity at port. Yet, whereas an increase in A’s shares in T reduces B’s output, it enhances A’s supply, because of their downward indirect effect on the integrated terminal fees. As a whole, the port throughput is negatively affected by terminal fees, but a stronger integration with A (captured by shares *s*) expands the total number of containers handled at the infrastructure.^4^ Inserting (2) into (1) yields first-stage gross profits, ${v}_{A}(f)$ and ${v}_{B}(f)$. In order to get the stage equilibrium, T maximizes the value function ${v}_{T}$ with respect to the terminal fee, leading to the equilibrium fee:

3 $$f=\frac{2\alpha (16-22s+17{s}^{2})}{64-108s+100{s}^{2}-11{s}^{3}}$$

As specified in Appendix A.2, the integrated terminal fee is inherently affected by market size. Modifications of the market size, as in the case of a decreasing $\alpha$, imply lower terminal fees *f*: as such, demand shocks impact the strategies of the terminal T, independently from the ownership structure. Yet, the effect of A’s shares on $f$ is more subtle. In case of terminal fees in the form of linear wholesale prices (i.e., ruling out two-part tariffs), a *tunnelling* effect arises (Brito et al. 2016; LaPorta et al. 2000). This effect deals with the carriers’ ability to ‘tunnel’ value of terminal operations from *passive* shareholders. Other things being equal, when vertically integrated, T considers A’s objectives by reducing its terminal fee (that is, A’s input costs). However, this implies a reduction in dividends for non-shipping shareholders (i.e., those not gaining from lower fees). For $s<\overline{s }$, seeking for additional revenues from liner B prevails over future savings on input costs (i.e., the tunnelling effect is rather weak), and T would raise the terminal fee. Contrarily, for $s\ge \overline{s }\cong 0.46$, increasing A’s control over T contributes to lower terminal fees, thus enhancing the tunnelling effect (again, see details in Appendix A.2). By inserting (3) into (1)–(2), we state the following:

##### Result 1

*Increasing (decreasing) the share of a carrier in terminals implies: (i) a larger (smaller) throughput and gross profits for integrated (non-integrated) companies, and (ii) a greater total throughput at port. Yet, equilibrium figures depend on the market size, as adverse demand shocks reduce each outcome:*

4 $${q}_{A}=\frac{2\alpha \left(2s-5{s}^{3}-4\right)}{11{s}^{3}-100{s}^{2}+108s-64} \quad {q}_{B}=\frac{\alpha \left(7{s}^{3}-22{s}^{2}+20s-8\right)}{11{s}^{3}-100{s}^{2}+108s-64} \quad Q=\frac{\alpha \left(24s-3{s}^{3}-22{s}^{2}-16\right)}{11{s}^{3}-100{s}^{2}+108s-64}$$

5 $${v}_{A}=\frac{8{\alpha }^{2}\left(12+20s-121{s}^{2}+262{s}^{3}-249{s}^{4}+132{s}^{5}-11{s}^{6}\right)}{{\left(11{s}^{3}-100{s}^{2}+108s-64\right)}^{2}} \quad {v}_{B}=\frac{3{\alpha }^{2}{\left(7{s}^{3}-22{s}^{2}+20s-8\right)}^{2}}{2{\left(11{s}^{3}-100{s}^{2}+108s-64\right)}^{2}}$$

From Result 1, market size (measured by $\alpha$) has a straightforward impact on equilibrium outcomes, as either demand booms or slumps proportionally change quantities and gross profits. Instead, the intensity of the vertical integration between carriers and terminal operators (that is, A’s control over T in the model) has a twofold impact on carriers’ containers transported and gross profits. Firstly, a direct effect exists—due to the *double marginalization* in vertically related industries (in general, see Motta 2004)—for which increasing vertical-control means getting closer to a complete integration between carriers and terminal operators, inducing a more efficient supply chain (see also Riordan 2008 and Chen 2001). Secondly, an indirect and additional output-expanding effect occurs, due to the tunnelling effect, i.e., reduced fees charged to integrated carriers). In fact, the interplay leads to more container volumes supplied in the port, since the expansion of the integrated carriers’ output counterweighs the declining supply of non-integrated ones. Overall, that result extends to be in favour of the integrated carrier’s gross profits.^5^

#### Merger game

**Fig. 2:**
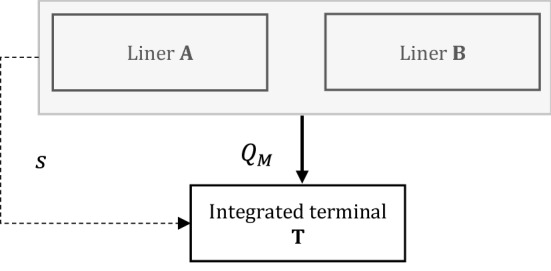
Merger between A and B with an integrated terminal only

We now assume that carriers A and B merge into a new entity M. As a result, the inverse market demand is ${p}_{M}=\alpha -{Q}_{M}$, where ${Q}_{M}={q}_{A,M}+{q}_{B,M}$ indicates total containers supplied by the new entity (Fig. 2).^6^ For the sake of simplicity, we rule out bargaining steps between the two carriers, therefore the incentive to merge is only related to larger prospective gross profits vis à vis the pre-merger status quo. We also consider the same timing for the pre-merger game. By following standard literature on asset-based horizontal mergers (e.g., Motta and Vasconcelos 2005; Perry and Porter 1985), we assume that carriers benefit from asset-specific efficiency gains, i.e., based on the aggregation of capacity endowments (see also Crotti et al. 2020). In particular, following McAfee and Williams (1992), each carrier enjoys a doubling in capacity with respect to the pre-merger case, i.e., ${k}_{A}+{k}_{B}={k}_{M}=2$. Thus, the total individual costs would be equal to $C\left({q}_{i,M}\right)={\left({q}_{i,M}\right)}^{2}/2{k}_{M}$, for $i=A,B$. As in the pre-merger case, the two-stage market equilibrium is derived by backward induction.

For a given terminal fee ${f}^{M}$, in the second stage the merging carriers maximize joint gross profits:

6 $${v}_{M}\left({q}_{A,M},{q}_{B,M};{f}^{M}\right)={\pi }_{A}^{M}+{\pi }_{B}^{M}+s{\pi }_{T}^{M}={p}_{M}\left({q}_{A,M}+{q}_{B,M}\right)-\frac{{\left({q}_{A,M}\right)}^{2}}{2}-\frac{{\left({q}_{B,M}\right)}^{2}}{2}-(1-s){f}^{M}\left({q}_{A,M}+{q}_{B,M}\right)$$

Since the market is served by a single company, now the impact of A’s shares on T’s decisions is also internalized by B. By maximizing (6) with respect to post-merger quantity, each liner sets post-merger (symmetrical) second-stage TEUs is:

7 $${q}_{A,M}\left({f}^{M}\right)={q}_{B,M}\left({f}^{M}\right)\triangleq {q}_{M}\left({f}^{M}\right)=\frac{2}{9}\left(\alpha -{f}^{M}\left(1-s\right)\right)$$

where (as in the pre-merger scenario) increasing fees reduce supplied TEUs, while higher *s* expand them.^7^

Inserting (7) into (6) and maximizing the terminal operator’s value function yields first-stage terminal fees^8^:

8 $${f}^{M}=\frac{\alpha }{2-s}.$$

If market conditions are worsening due, for instance, to a demand shock ($\alpha$ tends to 0), T reduces the terminal fee to increase the number of containers. By contrast to the pre-merger case, the integrated terminal fee is always increasing with the share of A in T. The reason is that, since the merger implies a monopolistic case, T is not committed to reduce A’s input costs for competition purposes anymore, and the tunnelling effect tends to disappear. This outcome could have exemptions in the case, for instance, of subsidies, as discussed in Merck (2020).

Inserting (8) into (6)–(7), we state the following^9^:

##### Result 2

*A merger between a vertically integrated and a non-integrated carrier would entail the following equilibrium outputs and gross profits:*

9 $${Q}_{M}\triangleq 2{q}_{M}=\frac{4\alpha }{9(2-s)} \quad {v}_{M}=\frac{2{\alpha }^{2}}{9{\left(2-s\right)}^{2}}$$

*As in the pre-merger case, the stronger the integration (higher s), the larger the total throughput and gross profits. Instead, demand shocks (lower* $\alpha$ *) reduce the equilibrium throughput and profit.*

Result 2 remains prospective, because it only occurs if A and B have the right incentives to merge, which mostly depends on two questions: Would their joint gross profits be larger with respect to the pre-merger scenario? How could demand shocks affect the merger incentives?

In order to answer these questions, we consider three key elements related to the integrated terminal scenario: (i) the difference between post- and pre-merger joint gross profits, i.e., $\Delta {v}_{M}\triangleq {v}_{M}-\left({v}_{A}+{v}_{B}\right)$, (ii) the difference between post- and pre-merger total port throughput, i.e., $\Delta {Q}_{M}\triangleq {Q}_{M}-Q$, and (iii) the demand sensitivity of merger incentives, $\frac{\Delta {v}_{M}}{{\alpha }^{2}}\equiv \delta$, that is, an indicator capturing the impact of demand variations on the carriers’ gross profits. After some algebra, as specified in the Appendix A.7, we can summarize the results in the following:

##### Proposition 1

*A merger between vertically integrated and non-integrated carriers would only occur for relatively low (* $0\le s\le {\overline{s} }_{L}$ *) or high (* ${\overline{s} }_{H}\le s\le 1$ *) shares of the terminal, owned by the merging carriers. In the case of relatively low (high) shares: (i) the merger induces a smaller (greater) port throughput than the pre-merger setting; and (ii) for either very low or very high vertical integration, merger incentives are larger but also more sensitive to demand shocks.*

**Fig. 3:**
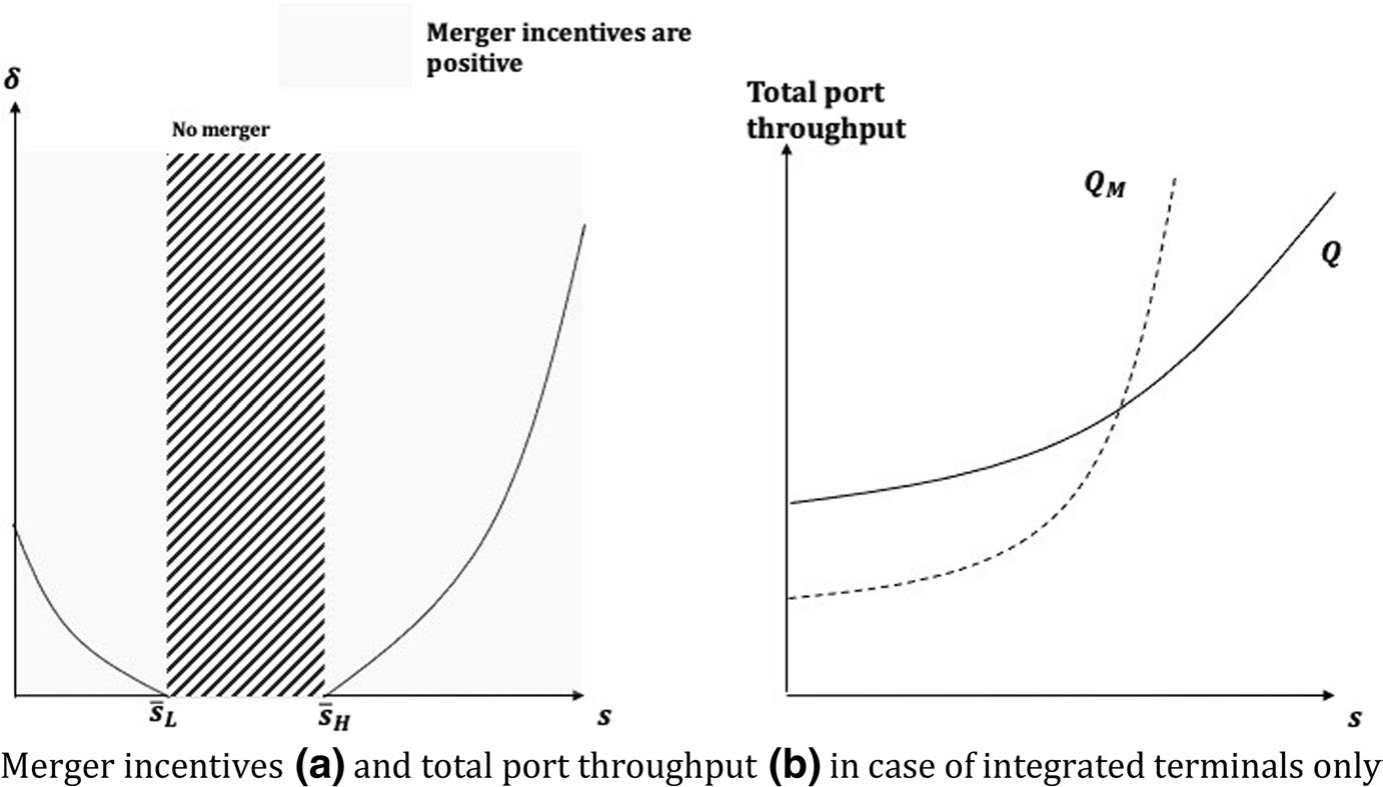
Merger incentives (**a**) and total port throughput (**b**) in case of integrated terminals only

As depicted in Fig. 3, the results in Proposition 1 can be explained as follows. If the carriers own a relatively small share in T (i.e., $s\le {\overline{s} }_{L}$), they gain smaller gross profits than in the pre-merger situation because the horizontal integration between carriers has the standard effect of reducing the total quantity of containers transported to or from the port, i.e., $\Delta {Q}_{M}<0$(Fig. 3.b). In that region of parameter, the incentive to merge is positive but their sensitivity to demand conditions (captured by $\delta$) is increasing when *s* tends to zero, i.e., in case the integration between carriers and integrated terminals is close to null (Fig. 3a). When holding relatively high shares (i.e., $s\ge {\overline{s} }_{H}$), the merger incentives are still positive and, in that case, are sustained by the fact that a larger control over the integrated terminal exists and increases the merging carriers’ supply ($\Delta {Q}_{M}>0$) as in Result 2. In Fig. 3a, shows how the merger incentives are more sensitive to demand shocks when the industry is rather fully integrated, that is, when *s* is close to 1. Essentially, this result states a sort of duality as the impact from mergers might be more sensitive to variations in market demand conditions for either weakly or strongly vertically integrated shipping industries. Finally, whereas the post-merger terminal fees are always increasing in *s* (see Appendix A.5), instead for intermediate shares (i.e., ${\overline{s} }_{L}<s<{\overline{s} }_{H}$), the pre-merger terminal fees are at their highest (see Appendix A.2), thus the gains from merger turn out to be definitely negative, and a horizontal integration would never occur.

### Scenario with one integrated terminal and one non-integrated terminal

**Fig. 4:**
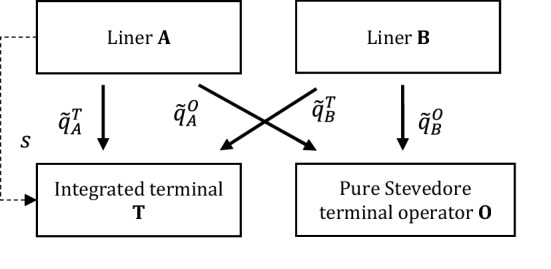
Liner shipping market with integrated and non-integrated terminals

In this alternative scenario, we consider the same baseline as in previous cases, except for the fact that now A and B are assumed to call at two terminals: a terminal T, integrated with container carrier A, and a non-integrated terminal O, completely separated from the carriers. (Fig. 4). We are then facing a case of two upstream operators, competing in setting terminal fees. As a result, the inverse demand function rewrites as follows: $\tilde{p }=\alpha -\tilde{Q }$, where $\tilde{Q }\triangleq {\tilde{Q }}^{T}+{\tilde{Q }}^{O}=\left({\tilde{q }}_{A}^{T}+{\tilde{q }}_{B}^{T}\right)+\left({\tilde{q }}_{A}^{O}+{\tilde{q }}_{B}^{O}\right)$ is the total port throughput, including the A’s and B’s supply either at the integrated and/or the non-integrated terminal, respectively. When calling at the terminals, the carriers must pay a fee to each of them, i.e., ${\tilde{f }}_{T}$ and ${\tilde{f }}_{O}$, and still bear costs with decreasing returns, i.e., $C\left({\tilde{q }}_{i}^{T},{\tilde{q }}_{i}^{O}\right)=\frac{{\left({\tilde{q }}_{i}^{T}\right)}^{2}+{\left({\tilde{q }}_{i}^{O}\right)}^{2}}{2{k}_{i}}$, for $i=A,B$.

#### Pre-merger game

The pre-merger game is solved by backward induction and for the second stage A and B set their own supply, given the terminal fees ${\tilde{f }}_{T}$ and ${\tilde{f }}_{O}$. Meanwhile, the carriers maximize gross profits so that:

10 $$\begin{aligned}&{\tilde{v }}_{A}\left({\tilde{q }}_{i}^{T},{\tilde{q }}_{i}^{O};{\tilde{f }}_{T},{\tilde{f }}_{O}\right)\triangleq {\stackrel{\sim }{\pi }}_{A}+s{\stackrel{\sim }{\pi }}_{T}=\tilde{p }\left({\tilde{q }}_{A}^{T}+{\tilde{q }}_{A}^{O}\right)-\frac{{\left({\tilde{q }}_{A}^{T}\right)}^{2}+{\left({\tilde{q }}_{A}^{O}\right)}^{2}}{2{k}_{i}}-{\tilde{f }}_{T}{\tilde{q }}_{A}^{T}-{\tilde{f }}_{O}{\tilde{q }}_{A}^{O}+s{\tilde{f }}_{T}\left({\tilde{q }}_{A}^{T}+{\tilde{q }}_{B}^{T}\right)\\ &{\tilde{v }}_{B}\left({\tilde{q }}_{i}^{T},{\tilde{q }}_{i}^{O};{\tilde{f }}_{T},{\tilde{f }}_{O}\right)\triangleq {\pi }_{B}=\tilde{p }\left({\tilde{q }}_{B}^{T}+{\tilde{q }}_{B}^{O}\right)-\frac{{\left({\tilde{q }}_{B}^{T}\right)}^{2}+{\left({\tilde{q }}_{B}^{O}\right)}^{2}}{2{k}_{i}}-{\tilde{f }}_{T}{\tilde{q }}_{B}^{T}-{\tilde{f }}_{O}{\tilde{q }}_{B}^{O}\end{aligned}$$

for $$i=A,B$$. Still assuming unit carriers’ capacity endowment ($${k}_{i}=1)$$, the ability for A to save input costs and recoup revenues from B through own control over T is once considered. In the second-stage, the maximization of carriers’ value functions in (10) yields their own supply at both the integrated and non-integrated terminal, as a function of respective terminal fees:

11 $$\begin{aligned}&{\tilde{q }}_{A}^{T}\left({\tilde{f }}_{T},{\tilde{f }}_{O}\right)=\frac{\alpha }{7}-\frac{{\tilde{f }}_{T}}{7}\left(4-\frac{13}{3}s\right)+\frac{3{\tilde{f }}_{O}}{7}\\&{\tilde{q }}_{B}^{T}\left({\tilde{f }}_{T},{\tilde{f }}_{O}\right)=\frac{\alpha }{7}-\frac{{\tilde{f }}_{T}}{7}\left(4+\frac{s}{3}\right)+\frac{3{\tilde{f }}_{O}}{7}\\&{\tilde{q }}_{A}^{O}\left({\tilde{f }}_{T},{\tilde{f }}_{O}\right)=\frac{\alpha }{7}+\frac{{\tilde{f }}_{T}}{7}\left(3-\frac{8}{3}s\right)-\frac{4{\tilde{f }}_{O}}{7}\\&{\tilde{q }}_{B}^{O}\left({\tilde{f }}_{T},{\tilde{f }}_{O}\right)=\frac{\alpha }{7}+\frac{{\tilde{f }}_{T}}{7}\left(3-\frac{s}{3}\right)-\frac{4{\tilde{f }}_{O}}{7}\end{aligned}$$

Considering the effect of terminal fees on traffic, since the two terminals compete on price, as expected, increasing the fees set by either T or O reduce the carriers’ supply at own terminal and increase that at the rival one. Actually, setting higher terminal fees tends to divert the supplied capacity towards other port facilities. Interestingly, if A’s shares increase, only the container volume by carrier A at the integrated terminal would increase, while all the other outputs are reduced. Therefore, in this scenario, the major effect of larger shares *s* is to divert A’s supply towards the integrated terminal, leaving the pure stevedore-managed terminal with fewer containers to be handled. As for the total throughput at terminals, we get ${\tilde{Q }}^{T}\left({\tilde{f }}_{T},{\tilde{f }}_{O}\right)=\frac{2}{7}\left(\alpha +3{\tilde{f }}_{O}-2{\tilde{f }}_{T}(2-s)\right)$ and ${\tilde{Q }}^{O}\left({\tilde{f }}_{T},{\tilde{f }}_{O}\right)=\frac{1}{7}\left(2\alpha +6{\tilde{f }}_{T}\left(1-\frac{s}{2}\right)-8{\tilde{f }}_{O}\right)$.

Despite the fact that a shock in demand (lower $\alpha$) reduces the overall throughput at both the terminals, increasing integrated terminal fees ${\tilde{f }}_{T}$ would reduce (expand) the total throughput at the integrated (pure stevedore managed) terminal, whereas higher A’s shares have the opposite effect.^10^

Inserting (11) into (10) and maximizing the terminal operators’ value functions: ${\tilde{v }}_{T}=s{\tilde{v }}_{A}\left({\tilde{f }}_{T},{\tilde{f }}_{O}\right)+(1-s){\tilde{f }}_{T}\left({\tilde{q }}_{A}^{T}\left({\tilde{f }}_{T},{\tilde{f }}_{O}\right)+{\tilde{q }}_{B}^{T}\left({\tilde{f }}_{T},{\tilde{f }}_{O}\right)\right)$ and ${\tilde{v }}_{O}={\stackrel{\sim }{\pi }}_{O}={\tilde{f }}_{O}\left({\tilde{q }}_{A}^{O}\left({\tilde{f }}_{T},{\tilde{f }}_{O}\right)+{\tilde{q }}_{B}^{O}\left({\tilde{f }}_{T},{\tilde{f }}_{O}\right)\right)$, the pre-merger integrated and non-integrated terminal fees are:

12 $$\begin{aligned}&{\tilde{f }}_{T}=\frac{2\alpha \left(972s-788{s}^{2}-693\right)}{11745s-10212{s}^{2}+1370{s}^{3}-6930}\\&{\tilde{f }}_{O}=\frac{\alpha (4914s-4389{s}^{2}+896{s}^{3}-2772)}{2\left(11745s-10212{s}^{2}+1370{s}^{3}-6930\right)}\end{aligned}$$

Similar to a single integrated terminal scenario, both terminal fees are affected by demand shocks (decreasing $\mathrm{\alpha }$). As a whole, the integrated terminal fee is always higher than the non-integrated terminal one, i.e., ${\tilde{f }}_{T}>{\tilde{f }}_{O}$. For increasing values of *s* (the level of A’s vertical integration with T), the fee of the non-integrated terminal ${\tilde{f }}_{O}$ is always lowered to attract more containers, because a larger share of carrier A in terminal T induces lower output at the non-integrated terminal (as in Appendix B.1).

By contrast, in the present scenario with competition between integrated and pure stevedore-managed terminals, the pricing strategy of T (variously integrated with A) is non-monotonic. For $s<\tilde{s }\cong 0.70$, the integrated terminal fee is raised by A’s shares, as saving input costs are less relevant than recouping revenues from B. Contrarily, for $s\ge \tilde{s }$, again the tunnelling effect offsets the importance of additional revenues, and T looks after A’s interests by lowering own fee.^11^ By using the terminal fees in (12), we state the following:

##### Result 3

*In the presence of both an integrated and a non-integrated terminal, the total throughput handled at the former is greater than that at the latter. A stronger vertical integration between A and T implies (i) more TEUs supplied, and (ii) larger gross profits for the integrated liner. Still in this case, demand shocks would reduce equilibrium figures:*

13 $$\begin{aligned}&{\tilde{q }}_{A}^{T}=\frac{\alpha \left(1524s-705{s}^{2}-1052{s}^{3}-1584\right)}{2(1370{s}^{3}-10212{s}^{2}+11745s-6930)}\quad {\tilde{q }}_{A}^{O}=\frac{\alpha \left(1635s-1578{s}^{2}+502{s}^{3}-792\right)}{1370{s}^{3}-10212{s}^{2}+11745s-6930}\\ & {\tilde{q }}_{B}^{T}=\frac{\alpha \left(3372s-3297{s}^{2}+916{s}^{3}-1584\right)}{2(1370{s}^{3}-10212{s}^{2}+11745s-6930)} \quad {\tilde{q }}_{B}^{O}=\frac{\alpha \left(1173s-930{s}^{2}+10{s}^{3}-792\right)}{1370{s}^{3}-10212{s}^{2}+11745s-6930}\\ &{\tilde{Q }}^{T}=\frac{\alpha \left(2448s-2001{s}^{2}+68{s}^{3}-1584\right)}{1370{s}^{3}-10212{s}^{2}+11745s-6930} \quad {\tilde{Q }}^{O}=\frac{4\alpha \left(702s-627{s}^{2}+128{s}^{3}-396\right)}{1370{s}^{3}-10212{s}^{2}+11745s-6930}\end{aligned}$$

14 $$\begin{aligned}&{\tilde{v }}_{A}=\frac{{\alpha }^{2}\left(25090560-67155264s+89127036{s}^{2}-42216120{s}^{3}-7912845{s}^{4}+22475688{s}^{5}-3288736{s}^{6}\right)}{8{\left(1370{s}^{3}-10212{s}^{2}+11745s-6930\right)}^{2}}\\& {\tilde{v }}_{B}=\frac{{\alpha }^{2}\left(25090560-90573120s+163952028{s}^{2}-163739232{s}^{3}+95198643{s}^{4}-25422312{s}^{5}+2591648{s}^{6}\right)}{8{\left(1370{s}^{3}-10212{s}^{2}+11745s-6930\right)}^{2}}\end{aligned}$$

Here it is important to note that, given the vertical relationship with T, the liner company A’s quantity of TEUs handled at the integrated terminal is always greater than that at the pure stevedore-managed one, i.e., ${\tilde{q }}_{A}^{T}>{\tilde{q }}_{A}^{O}$, while the opposite occurs for B, as ${\tilde{q }}_{B}^{O}>{\tilde{q }}_{B}^{T}$. Overall, the operator T would handle a larger throughput than O, and this output-based dominance by T is accrued for increasing A’s control over T.^12^

#### Merger game

**Fig. 5:**
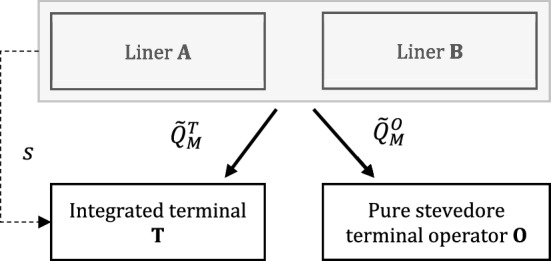
Merger between A and B with integrated and non-integrated terminals

We finally consider the incentive for A and B to merge. Hence, in this case we study a situation where, in case of upstream competition between terminals, the downstream interplay between carriers is eliminated by a horizontal merger (Fig. 5). Still assuming asset-based efficiencies by aggregating carriers’ capacity, when merging A and B decide their own TEUs supply at each terminal, now acting as a single entity and maximizing joint gross profits in the second stage. The inverse demand function is: ${\tilde{p }}_{M}=\alpha -{\tilde{Q }}_{M}$, where ${\tilde{Q }}_{M}\triangleq {\tilde{Q }}_{M}^{T}+{\tilde{Q }}_{M}^{O}=\left({\tilde{q }}_{A,M}^{T}+{\tilde{q }}_{B,M}^{T}\right)+\left({\tilde{q }}_{A,M}^{O}+{\tilde{q }}_{B,M}^{O}\right)$. ${\tilde{Q }}_{M}$ is the total post-merger throughput. As for carriers’ costs, they still encompass a double capacity by merger, conducing to $C\left({q}_{i,M}^{T},{q}_{i,M}^{O}\right)=\frac{{\left({q}_{i,M}^{T}\right)}^{2}+{\left({q}_{i,M}^{O}\right)}^{2}}{2{k}_{M}}$, for $i=A,B$, and where ${k}_{M}=2$.

The related joint gross profits are:

15 $${\tilde{v }}_{M}\left({\tilde{q }}_{i,M}^{T},{\tilde{q }}_{i,M}^{O};{\tilde{f }}_{M}^{T},{\tilde{f }}_{M}^{O}\right)={\stackrel{\sim }{\pi }}_{A,M}+{\stackrel{\sim }{\pi }}_{B,M}+s{\stackrel{\sim }{\pi }}_{T}={\tilde{p }}_{M}\sum_{i=A,B}\left({\tilde{q }}_{i,M}^{T}+{\tilde{q }}_{i,M}^{O}\right)-\sum_{i=A,B}\frac{{\left({q}_{i,M}^{T}\right)}^{2}+{\left({q}_{i,M}^{O}\right)}^{2}}{4}-(1-s){\tilde{f }}_{M}^{T}\sum_{i=A,B}{q}_{i,M}^{T}$$

Maximizing (15) we get second-stage quantities:

16 $$\begin{aligned}&{\tilde{q }}_{A,M}^{T}\left({\tilde{f }}_{M}^{T},{\tilde{f }}_{M}^{O}\right)={\tilde{q }}_{B,M}^{T}\left({\tilde{f }}_{M}^{T},{\tilde{f }}_{M}^{O}\right)\triangleq {\tilde{q }}_{M}^{T}\left({\tilde{f }}_{M}^{T},{\tilde{f }}_{M}^{O}\right)=\frac{1}{17}\left(2\alpha -18{\tilde{f }}_{M}^{T}\left(1-s\right)+16{\tilde{f }}_{M}^{O}\right)\\&{\tilde{q }}_{A,M}^{O}\left({\tilde{f }}_{M}^{T},{\tilde{f }}_{M}^{O}\right)={\tilde{q }}_{B,M}^{O}\left({\tilde{f }}_{M}^{T},{\tilde{f }}_{M}^{O}\right)\triangleq {\tilde{q }}_{M}^{O}\left({\tilde{f }}_{M}^{T},{\tilde{f }}_{M}^{O}\right)=\frac{1}{17}\left(2\alpha +16{\tilde{f }}_{M}^{T}\left(1-s\right)-18{\tilde{f }}_{M}^{O}\right)\end{aligned}$$

For the merger case, the impact of terminal fees and the shares of carrier A’s container volumes transported at the terminal T and O display opposite directions. As expected, the competitive price between the terminal operators implies that increasing the fees of the integrated terminal T (non-integrated terminal O) would reduce (raise) the total output at the integrated (non-integrated) terminal. In case of larger shares of carrier A in terminal T, an increasing number of containers are shifted towards the integrated terminal, to the detriment of the non-integrated terminal.^13^ Inserting (16) into (15) yields first-stage merging carriers’ gross profits, and then, in order to get the first-stage equilibrium, T maximizes ${\tilde{v }}_{T}^{M}=s{\tilde{v }}_{M}\left({\tilde{f }}_{M}^{T},{\tilde{f }}_{M}^{O}\right)+(1-s){\tilde{f }}_{M}^{T}{\tilde{q }}_{M}^{T}\left({\tilde{f }}_{M}^{T},{\tilde{f }}_{M}^{O}\right)$, and O maximizes: ${\tilde{v }}_{O}^{M}={\stackrel{\sim }{\pi }}_{O}^{M}=$ ${\tilde{f }}_{M}^{O}{\tilde{q }}_{M}^{O}\left({\tilde{f }}_{M}^{T},{\tilde{f }}_{M}^{O}\right)$, yielding terminal fees:

17 $${\tilde{f }}_{M}^{T}=\frac{13\alpha }{130-49s} \quad and \quad {\tilde{f }}_{M}^{O}=\frac{\alpha \left(26-17s\right)}{2\left(130-49s\right)}$$

Similarly to a single integrated terminal, the main effect of increasing shares owned by A (now merged with B) is to raise the integrated terminal’s fee, whereas the pure stevedore-managed terminal fee would be lowered by O to capture more demand. Even for a single carrier in the presence of two competing terminals, the tunnelling effect tends to disappear, as saving input costs is a second-order driver than channelling fees to the final consumers. Nevertheless, the existence of negative demand conditions (shrinking $\alpha$) would lower both terminal fees.^14^

Finally, inserting (17) into (15)–(16) we state the following:

##### Result 4

*In the presence of both a integrated and a non-integrated terminal, the post-merger total throughput at both the terminals, and the merged carriers’ gross profits are, respectively:*

18 $$\begin{aligned}&{\tilde{Q }}_{M}^{T}\triangleq 2{\tilde{q }}_{M}^{T}=\frac{468\alpha }{2210-833s} \quad {\tilde{Q }}_{M}^{O}\triangleq 2{\tilde{q }}_{M}^{O}=\frac{18\alpha \left(26-17s\right)}{17\left(130-49s\right)}\\&{\tilde{v }}_{M}=\frac{81{\alpha }^{2}\left(153{s}^{2}-884s+1352\right)}{34{\left(130-49s\right)}^{2}}\end{aligned}$$

*The vertical integration (higher s) induces larger (smaller) post-merger total throughput at the integrated (pure stevedore managed) terminal, but still greater merging carriers’ gross profits. Demand shocks (lower* $\alpha$ *) would reduce the equilibria.* 15

We then consider the incentives to merge for carriers, together with the related impact of demand shocks on merger gains and finally look at the effects of the horizontal integration on the overall port throughput. Again, by defining $\Delta {\tilde{v }}_{M}\triangleq {\tilde{v }}_{M}-\left({\tilde{v }}_{A}+{\tilde{v }}_{B}\right)$, $\Delta {\tilde{Q }}_{M}\triangleq {\tilde{Q }}_{M}-\tilde{Q }$, and $\frac{\Delta {\tilde{v }}_{M}}{{\alpha }^{2}}\equiv \stackrel{\sim }{\delta }$, we can conclude that:

##### Proposition 2

*In the presence of both integrated and pure stevedore-managed terminals, (i) a merger between vertically integrated and non-integrated carriers will always occur in this model; (ii) the post-merger throughput at the integrated terminal is larger than the pre-merger case only for large enough integrations (* $$s>0.33);$$ *(iii) the post-merger supply at the non-integrated terminal is always lower than before the merger; (iv) the overall port throughput is reduced by the merger; and finally (v) higher incentives to merge are present for relatively low vertical integration, whereas those incentives are also more harmed by demand shocks.*

**Fig. 6:**
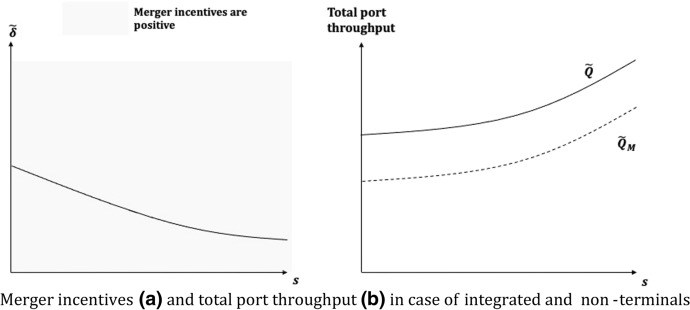
Merger incentives (**a**) and total port throughput (**b**) in case of integrated and non-integrated terminals

Looking at Fig. 6, the results of Proposition 2 are motivated as follows. By comparing the post- and pre-merger joint gross profits, it is possible to check that $\Delta {\tilde{v }}_{M}$ is a strictly positive function for any value of *s*. Thus, differently from what was obtained in the previous scenario, the presence of two terminals (one integrated and one non-integrated) implies that a merger between A and B would always occur. Again, the indicator $\stackrel{\sim }{\delta }$ helps us to investigate the relative impact of demand shocks on merger incentives. Actually, differently from Proposition 1, here the horizontal integration conveys larger gains for relatively low private terminal’s shares held by the merging carriers. In that case, potential demand shocks have a stronger (downward) marginal effect on profits, thus reducing the prospective benefit for merging companies.

By contrast, although merger incentives are weaker, for very high vertical integration the negative marginal effect of demand shocks on merger gains would be lower (Fig. 6.a). As for the effect of the merger on terminal throughput, the presence of a pure stevedore-managed terminal—whose level of output is always lower than the integrated one (i.e., ${\tilde{Q }}_{M}^{T}>{\tilde{Q }}_{M}^{O}$)—determines the ability of merging carriers to divert the most part of demanded TEUs towards the integrated terminal, thus enlarging the profitability of the merger. In fact, $\Delta {\tilde{Q }}_{M}^{T}>0$ for $s>0.33$, while $\Delta {\tilde{Q }}_{M}^{O}<0$ for any *s*. In general, the total throughput at port is reduced by the merger, as the carriers would redirect most of the demanded TEUs towards the integrated terminal, i.e., $\Delta {\tilde{Q }}_{M}\triangleq {\tilde{Q }}_{M}-\tilde{Q }<0$ for any *s* (Fig. 6.b).^16^

## Discussion and concluding remarks

The analysis performed above is a first attempt to assess the impact of demand variations on the attitude of industry players to increase their level of integration and the effects of this in terms of market (i.e., concentration and port activity) and welfare. Our study is particularly important given the demand shock caused by the Covid-19 pandemic, firstly through an unprecedent demand reduction (due to local and regional lockdowns) and then through an unexpected bounce-back for most markets. Within this framework, demand shocks represent incentives to pursue vertical integration strategies, in the presence of a plurality of terminals within the port, but they generate different trade-offs, considering either the social welfare or the public viewpoint.

From a social welfare point of view, mergers do not always imply an increase in port throughput: integrations between carriers and terminals might improve the competitive position of the private operators promoting the merger. But this is often not the case from a public point of view: there is a difference in interests and, hence, appreciation of the effects of mergers among the stakeholders involved (e.g., regulators, port authorities, terminal operators, and shipping companies). Moreover, vertical integrations do have an impact on terminal fees, often reducing them: this is one of the advantages of pushing integration-related behaviours. Furthermore, the proposed analysis has underlined how the coexistence of integrated and pure stevedore-operated terminals at port could determine different outcomes in terms of total throughput and carriers’ gross profits.

In Table 1, the main results according to the two studied scenarios are summarized. In particular, the presence of integrated terminals would likely imply mergers between carriers only if the carriers’ shareholdings into terminals are either relatively weak ($s\le {\overline{s} }_{L}\equiv 0.41$) or strong ($s\ge {\overline{s} }_{H}\equiv 0.73$), resulting into market equilibria where merging carriers gain large profits, but the total throughput (and thus consumer welfare) is expanded only in the case of significant vertical integration ($s>0.45$). Introducing non-integrated terminals (managed by pure stevedores) provides a scenario where mergers are always likely to occur, but where the total throughput might decrease to the detriment of final consumers. In terms of the impact of demand variations on the incentives to merge, the two scenarios provide notable results. In the first scenario, the largest effect, caused by demand changes on merger profits, occurs for either null or total integration between carriers and terminal. This implies that, besides zero shareholdings of a terminal, the decision to largely invest into a terminal might also convey potential higher gains from merger. By contrast, in the second scenario, owning smaller shareholdings entails a stronger impact of demand variations on merger profits, while a rather complete integration would imply a lower sensitivity, and therefore weaker merger incentives.

**Table 1:** Summary of results

Scenario	Positive merger incentives	Larger post-merger throughput	Merger sensitivity to demand variations	
			Strong	Weak
Only one integrated terminal	$s\le {\overline{s} }_{L}\equiv 0.41$ and $s\ge {\overline{s} }_{H}\equiv 0.73$	$s>0.45$	$s\to 0$ and $s\to 1$	$s\to {\overline{s} }_{L}\equiv 0.41$ and $s\to {\overline{s} }_{H}\equiv 0.73$
One integrated terminal and one non-integrated terminal	Always ($\forall s$)	Never ($\nexists s$)	$s\to 0$	$s\to 1$

For a port authority, maximizing social welfare (carriers’ profits plus consumer surplus), which setting would convey the best configuration is a topic that deserves further investigation, especially with regard to the potential long-term negative demand conditions in the industry. On the one hand, it would be interesting to assess the expected social welfare when comparing the two scenarios, thus considering whether the trade-off between higher profits and smaller throughput would end up to a larger social welfare. On the other hand, by contrasting the two port configurations, understanding in which case merger projects are able to raise or reduce the status quo (pre-merger) social welfare would be of key relevance.

For a public port authority, the internal organisation of the port (e.g., the presence of more than one terminal, or the ability to promote both integrated and non-integrated terminals) could affect the overall social welfare generated by vertical integration strategies, particularly if linked with demand variations. As such, an assessment of the consequences of such strategic solutions on port throughput and fees (e.g., public goals vs. private revenues) appears as necessary in all different circumstances. Our results indeed suggest that the co-existence of integrated and non-integrated terminals do not always help in achieving higher levels of throughput (particularly whenever the integrated carriers could enjoy other kinds of advantages). Thus, our results imply that in order to achieve public goals (e.g., increase tax collection, employment levels, etc.), port management bodies should introduce proper monitoring systems as well as effective regulation so as to promote an optimal level of service. Similarly, competition authorities are often involved in the evaluation of mergers and acquisitions, as well as in assessing the market impact of other strategic agreements that could affect competition, market concentration, and generally on service characteristics. Eventually, as in the case of the Belt and Road Initiative (Ferrari and Tei 2020), maritime-related integration strategies often entail geopolitical challenges as well. These latter aspects (i.e., port social welfare in case of integrated carriers and related regulatory tools) certainly need further assessment and they will be part of an already planned future research development.

The paper shows how demand shocks—as the one experienced during the Covid-19 pandemic—and market organisation (i.e., non-integrated vs. either horizontally or vertically integrated operators) create different sets of conditions to further push towards market integrations, modifying the willingness to pursue M&A and allowing for alternative strategic behaviours. Authors are aware of the limitations of the study. In order to improve the robustness of the achieved results, future steps of the analysis will include a numerical testing, using a real case study for better fine-tuning the model and the possibility to increase the number of possible scenarios (e.g., plurality of terminals with different market conditions, alliances, and alternative tariff strategies) so as to better evaluate the several situations actually occurring in the market.

## Appendix A—Scenario with an integrated terminal only

### A.1 Second-stage equilibrium in the pre-merger game.

The quantity-setting equilibrium is derived by maximizing each carrier’s value function in (1) with respect to shipped TEUs. The first-order conditions (FOCs), $\partial {v}_{i}/\partial {q}_{i}=0$ for $i=A,B$, give a system of best-response functions: ${q}_{A}\left({q}_{B}\right)=\frac{1}{3}\left(\alpha -{q}_{B}-f\left(1-s\right)\right)$ and ${q}_{B}\left({q}_{A}\right)=\frac{1}{3}\left(\alpha -{q}_{A}-f\right)$, showing that carriers compete in *strategic substitutes*, i.e., players react to rivals’ increasing TEUs by reducing own supply. Related second-order conditions (SOCs) for local maxima of concave functions are fulfilled, as $\frac{{\partial }^{2}{v}_{i}}{\partial {{q}_{i}}^{2}}=-\frac{1}{3}<0$, for $i=A,B$. The solution of the above system yields second-stage outputs, as in (2). By comparative statics, we check that increasing fees reduce both carriers’ quantity supplied: $\frac{\partial {q}_{A}\left(f\right)}{\partial f}=-\frac{1}{4}+\frac{3s}{8}<0$ for $s<\frac{2}{3}$, and $\frac{\partial {q}_{B}\left(f\right)}{\partial f}=-\frac{1}{4}-\frac{s}{8}<0$ for $i=A, B$. As for the effect of increasing A’s shares, $\frac{\partial {q}_{A}\left(f\right)}{\partial s}=\frac{3f}{8}>0$ means that A’s supply goes up, while, for liner B, $\frac{\partial {q}_{B}\left(f\right)}{\partial s}=-\frac{f}{8}<0$ implies less TEUs shipped by B. Overall, the effect of increasing terminal fees on the total quantity of TEUs at port is negative, as $\frac{\partial Q\left(f\right)}{\partial f}=-\frac{1}{2}+\frac{s}{4}<0$ for $\forall s$. For rising A’s shares, this effect is reverted, i.e., $\frac{\partial Q\left(f\right)}{\partial s}=\frac{f}{4}>0$.

### A.2 First-stage equilibrium in the pre-merger game

The first-stage equilibrium is derived by maximizing ${v}_{T}=s{v}_{A}f+(1-s)f\left({q}_{A}\left(f\right)+{q}_{B}\left(f\right)\right)$ with respect to $f$, i.e., $\frac{\partial {v}_{T}}{\partial f}=\frac{\alpha }{2}-f+\frac{17}{32}\alpha {s}^{2}-\frac{25}{16}{s}^{2}f+\frac{11}{64}{s}^{3}f-\frac{11}{16}\alpha s+\frac{27}{16}sf=0$. Solving for $f$ yields the equilibrium terminal fee in this pre-merger scenario (with an integrated terminal only) as in (3). Searching for local maxima, the second derivative $\frac{{\partial }^{2}{v}_{T}}{\partial {f}^{2}}=\frac{11}{64}{s}^{3}-\frac{25}{16}{s}^{2}+\frac{27}{16}s-1$ is negative, as required. By simple comparative statics, we first check that the terminal fee is always positive (i.e., it gives positive values for any $\alpha$ and/or *s*). As a result, a lower $\alpha$ means lower fees as well. As for the effect of A’s shares, $\frac{\partial f}{\partial s}=\frac{2\alpha \left(187{s}^{4}-484{s}^{3}+892{s}^{2}-1024s+320\right)}{{\left(11{s}^{3}-100{s}^{2}+108s-64\right)}^{2}}$. As the denominator is positive, the sign of the derivative depends on the numerator, which is positive (negative) for $s<\left(>\right) \overline{s }\cong 0.46$. Therefore, a larger (smaller) vertical integration causes lower (higher) terminal fee.

### A.3 Equilibrium outcomes in the pre-merger game

The impact of A’s shares on equilibrium figures is evaluated by comparative statics. Since $\frac{\partial {q}_{A}}{\partial s}=\frac{8\alpha \left(125{s}^{4}-281{s}^{3}+323{s}^{2}-200s+76\right)}{{\left(11{s}^{3}-100{s}^{2}+108s-64\right)}^{2}}>0$ and $\frac{\partial {q}_{B}}{\partial s}=-\frac{2\alpha \left(229{s}^{4}-536{s}^{3}+728{s}^{2}-608s+208\right)}{{\left(11{s}^{3}-100{s}^{2}+108s-64\right)}^{2}}<0$ for $\forall s$, increasing shares by A expands its output, and in turn reduces B’s one. As a whole, $\frac{\partial Q}{\partial s}=\frac{2\alpha \left(271{s}^{4}-588{s}^{3}+564{s}^{2}-192s+96\right)}{{\left(11{s}^{3}-100{s}^{2}+108s-64\right)}^{2}}>0$ implies that a larger integration induces a greater TEUs supply at port. Regarding gross profits, the impact of A’s shares on its profits is the following: $\frac{\partial {v}_{A}}{\partial s}=\frac{16{\alpha }^{2}\left(374{s}^{7}-6237{s}^{6}+19173{s}^{5}-32250{s}^{4}+33370{s}^{3}-22548{s}^{2}+9064s-1936\right)}{{\left(11{s}^{3}-100{s}^{2}+108s-64\right)}^{3}}>0$, while that on B’s profits is: $\frac{\partial {v}_{B}}{\partial s}=-\frac{6{\alpha }^{2}\left(1603{s}^{7}-8790{s}^{6}+21468{s}^{5}-32824{s}^{4}+33680{s}^{3}-22560{s}^{2}+9024s-1664\right)}{{\left(11{s}^{3}-100{s}^{2}+108s-64\right)}^{3}}<0$.

### A.4 Second-stage equilibrium in the post-merger game

The maximization of (7), i.e., $\partial {v}_{M}\left({q}_{A,M},{q}_{B,M};{f}^{M}\right)/\partial {q}_{i,M}=0$ yields two best-response functions:${q}_{i,M}\left({q}_{j,M}\right)=\frac{2}{5}\left(\alpha -2{q}_{j,M}-{f}^{M}(1-s)\right)$, for$i,j=A,B$. Solving the system leads to (8). As for the marginal effect of terminal fees and A’s shares, we get that: $\frac{\partial {q}_{M}\left({f}^{M}\right)}{\partial {f}^{M}}=-\frac{2}{9}(1-s)<0$ and $\frac{\partial {q}_{M}\left({f}^{M}\right)}{\partial s}=\frac{2}{9}{f}^{M}>0$.

### A.5 First-stage equilibrium in the post-merger game

The first-stage equilibrium is derived by maximizing ${v}_{T}^{M}=s{v}_{M}{f}^{M}+(1-s){f}^{M}\left({q}_{M}\left({f}^{M}\right)\right)$ with respect to ${f}^{M}$, i.e., $\frac{\partial {v}_{T}^{M}}{\partial {f}^{M}}=\frac{4}{9}{\left(1-s\right)}^{2}\left(\alpha -{f}^{M}(2-s)\right)=0$. Solving for ${f}^{M}$ yields the equilibrium terminal fee in the post-merger scenario in (9). The second-order condition is fulfilled, as $\frac{{\partial }^{2}{v}_{T}^{M}}{\partial {{f}^{M}}^{2}}=-\frac{4}{9}{\left(1-s\right)}^{2}\left(2-s\right)<0$. Higher (lower) $\alpha$ means higher (lower) fees, i.e., $\frac{\partial {f}^{M}}{\partial \alpha }=\frac{1}{2-s}>0$. Similarly, $\frac{\partial {f}^{M}}{\partial s}=\frac{\alpha }{{\left(2-s\right)}^{2}}>0$, thus post-merger terminal fees are always increasing in *s*.

### A.6 Equilibrium outcomes in the post-merger game

It is easily proven that $\frac{\partial {Q}_{M}}{\partial s}=\frac{4\alpha }{9{\left(2-s\right)}^{2}}>0$, $\frac{\partial {v}_{M}}{\partial s}=-\frac{4{\alpha }^{2}}{9{\left(s-2\right)}^{3}}>0$, therefore increasing A’s shares enhances post-merger equilibria. Conversely, since the level of demand is a multiplicative factor in each figure, demand shocks would lower them.

### A.7 Proof of Proposition 1

Starting from the gross profits’ gains by merger, by plotting the related function, we check that $\Delta {v}_{M}=\frac{{\alpha }^{2}\left(261{s}^{8}-11736{s}^{7}+59524{s}^{6}-123424{s}^{5}+151312{s}^{4}-118528{s}^{3}+59840{s}^{2}-18432s+2560\right)}{18{\left(11{s}^{4}-122{s}^{3}+308{s}^{2}-280s+128\right)}^{2}}$ is positive for either $s<{\overline{s} }_{L}\cong 0.41$ or $s>{\overline{s} }_{H}\cong 0.73$. Since $\Delta {Q}_{M}=\frac{\alpha \left(27{s}^{4}+100{s}^{3}-212{s}^{2}+144s-32\right)}{9\left(11{s}^{4}-122{s}^{3}+308{s}^{2}-280s+128\right)}<\left(>\right) 0$ for $s<\left(>\right) 0.44626$, therefore in the above former (latter) region the post-merger port throughput is lower (higher) than before the merger. As for the parameter $\delta$, its behaviour largely depends on which region of shares *s* we are considering. Actually, for $s<{\overline{s} }_{L}$, the fact that $\frac{\partial \delta }{\partial s}=\frac{4\left(16353{s}^{10}+110954{s}^{9}-1802388{s}^{8}+7912248{s}^{7}-17939440{s}^{6}+25514976{s}^{5}-24149568{s}^{4}+15582336{s}^{3}-6652416{s}^{2}+1751040s-231424\right)}{{9\left(11{s}^{4}-122{s}^{3}+308{s}^{2}-280s+128\right)}^{3}}<0$ implies that for values of *s* close to zero (very weak integration), the impact of demand variations on merger incentives is stronger. Similarly, as $\frac{\partial \delta }{\partial s}>0$ for any $s>{\overline{s} }_{H}$ means that a likewise fierce sensitivity to demand shocks is detected when the vertical integration is quite complete, i.e., for *s* tending to 1.

## Appendix B—Scenario with integrated and non-integrated terminals

### B.1 Second-stage equilibrium in the pre-merger game

Since each carrier sets the quantity of TEUs to be handled at both terminals, they maximize gross profits with respect to ${\tilde{q }}_{i}^{T}$ and ${\tilde{q }}_{i}^{O}$, i.e., $\partial {\tilde{v }}_{i}\left({\tilde{q }}_{i}^{T},{\tilde{q }}_{i}^{O};{\tilde{f }}_{T},{\tilde{f }}_{O}\right)/\partial {\tilde{q }}_{i}^{T}=0$, and $\partial {\tilde{v }}_{i}\left({\tilde{q }}_{i}^{T},{\tilde{q }}_{i}^{O};{\tilde{f }}_{T},{\tilde{f }}_{O}\right)/\partial {\tilde{q }}_{i}^{O}=0$, for $i=A,B$. Solving the following four-equation system of best-reply functions: ${\tilde{q }}_{A}^{T}\left({\tilde{q }}_{A}^{O}, {\tilde{q }}_{B}^{T},{\tilde{q }}_{B}^{O}\right)=\frac{1}{3}\left(\alpha -{\frac{2}{3}\tilde{q }}_{A}^{O}-{\tilde{q }}_{B}^{T}-{\tilde{q }}_{B}^{O}-{\tilde{f }}_{T}(1-s)\right)$, ${\tilde{q }}_{B}^{T}\left({\tilde{q }}_{B}^{O}, {\tilde{q }}_{A}^{T},{\tilde{q }}_{A}^{O}\right)=\frac{1}{3}\left(\alpha -{\frac{2}{3}\tilde{q }}_{B}^{O}-{\tilde{q }}_{A}^{T}-{\tilde{q }}_{A}^{O}-{\tilde{f }}_{T}\right)$, ${\tilde{q }}_{A}^{O}\left({\tilde{q }}_{A}^{T}, {\tilde{q }}_{B}^{T},{\tilde{q }}_{B}^{O}\right)=\frac{1}{3}\left(\alpha -{\frac{2}{3}\tilde{q }}_{A}^{T}-{\tilde{q }}_{B}^{T}-{\tilde{q }}_{B}^{O}-{\tilde{f }}_{O}\right)$, and ${\tilde{q }}_{B}^{O}\left({\tilde{q }}_{B}^{T}, {\tilde{q }}_{A}^{T},{\tilde{q }}_{A}^{O}\right)=\frac{1}{3}\left(\alpha -{\frac{2}{3}\tilde{q }}_{B}^{T}-{\tilde{q }}_{A}^{T}-{\tilde{q }}_{A}^{O}-{\tilde{f }}_{O}\right)$ yields the (13). By comparative statics, we prove that: $\frac{\partial {\tilde{q }}_{A}^{T}}{\partial {\tilde{f }}_{T}}=-\frac{1}{3}(1-s)<0$, $\frac{\partial {\tilde{q }}_{B}^{T}}{\partial {\tilde{f }}_{T}}=-\frac{1}{3}<0, \frac{\partial {\tilde{q }}_{A}^{O}}{\partial {\tilde{f }}_{O}}=-\frac{1}{3}<0$, and $\frac{\partial {\tilde{q }}_{B}^{O}}{\partial {\tilde{f }}_{O}}=-\frac{1}{3}<0$. Yet, $\frac{\partial {\tilde{q }}_{A}^{T}}{\partial s}=\frac{1}{3}{\tilde{f }}_{T}>0$. Since $\frac{\partial {\tilde{Q }}^{T}}{\partial {\tilde{f }}_{T}}=-\frac{4}{7}(2-s)<0$, $\frac{\partial {\tilde{Q }}^{T}}{\partial {\tilde{f }}_{O}}=\frac{6}{7}>0$, $\frac{\partial {\tilde{Q }}^{O}}{\partial {\tilde{f }}_{T}}=\frac{6}{7}{\tilde{f }}_{T}\left(1-\frac{s}{2}\right)>0$, $\frac{\partial {\tilde{Q }}^{O}}{\partial {\tilde{f }}_{O}}=-\frac{8}{7}<0$, and $\frac{\partial {\tilde{Q }}^{T}}{\partial s}=\frac{4}{7}{\tilde{f }}_{T}>0$, $\frac{\partial {\tilde{Q }}^{O}}{\partial s}=-\frac{3}{7}{\tilde{f }}_{O}<0$, thus increasing terminal fees benefit rivals’ terminal operators, while A’s shares push TEUs towards the integrated private terminal.

### B.2 First-stage equilibrium in the pre-merger game

The integrated terminal operator T (whose shares *s* are owned by the liner A) maximizes ${\tilde{v }}_{T}$ with respect to ${\tilde{f }}_{T}$, by imposing $\frac{\partial {\tilde{v }}_{T}}{\partial {\tilde{f }}_{T}}=0$, while the pure stevedore terminal company simply maximizes own profits, ${\tilde{v }}_{O}={\stackrel{\sim }{\pi }}_{O}$ with respect to ${\tilde{f }}_{O}$, i.e., $\frac{\partial {\tilde{v }}_{O}}{\partial {\tilde{f }}_{O}}=0$. Second-order conditions for local maxima are satisfied for both the problems, as $\frac{{\partial }^{2}{\tilde{v }}_{T}}{\partial {{\tilde{f }}_{T}}^{2}}=\frac{241}{441}{s}^{3}-\frac{508}{147}{s}^{2}+\frac{195}{49}s-\frac{16}{7}<0$ and $\frac{{\partial }^{2}{\tilde{v }}_{O}}{\partial {{\tilde{f }}_{O}}^{2}}=-\frac{16}{7}<0$ for any $s\le 1$. By inspecting (14), ${\tilde{f }}_{O}$ is lowered by increasing values of *s*, i.e., $\frac{\partial {\tilde{f }}_{O}}{\partial s}=-\frac{189\alpha \left(16598{s}^{4}-40120{s}^{3}+45513{s}^{2}-22308s+7920\right)}{2{\left(1370{s}^{3}-10212{s}^{2}+11745s-6930\right)}^{2}}<0$. Instead, ${\tilde{f }}_{T}$ is not monotonic in *s*, since $\frac{\partial {\tilde{f }}_{T}}{\partial s}=\frac{18\alpha \left(112340{s}^{4}-295920{s}^{3}+456276{s}^{2}-436128s+155925\right)}{{\left(1370{s}^{3}-10212{s}^{2}+11745s-6930\right)}^{2}}>\left(<\right) 0$ for $s<\left(>\right) \tilde{s }\cong 0.70$. In terms of difference between the two terminal fees, it is easy to check that ${\tilde{f }}_{T}-{\tilde{f }}_{O}=\frac{\alpha s\left(896{s}^{2}-1437s+1026\right)}{2(1370{s}^{3}-10212{s}^{2}+11745s-6930)}$ is positive for any *s*.

### B.3 Equilibrium outcomes in the pre-merger game

(i) The differences between TEUs supplied at both the integrated and non-integrated terminal by each liner are derived as follows: ${\tilde{q }}_{A}^{T}-{\tilde{q }}_{A}^{O}=-\frac{\alpha s\left(2056{s}^{2}-2451s+1746\right)}{2\left(1370{s}^{3}-10212{s}^{2}+11745s-6930\right)}>0$, while ${\tilde{q }}_{B}^{T}-{\tilde{q }}_{B}^{O}=\frac{\alpha s\left(896{s}^{2}-1437s+1026\right)}{2\left(1370{s}^{3}-10212{s}^{2}+11745s-6930\right)}<0$. Overall, ${\tilde{Q }}^{T}-{\tilde{Q }}^{O}=-\frac{\alpha s\left(580{s}^{2}-507s+360\right)}{1370{s}^{3}-10212{s}^{2}+11745s-6930}>0$. As for the impact of shares, we can also prove that $\frac{\partial {\tilde{Q }}^{T}}{\partial s}=\frac{207\alpha \left(16598{s}^{4}-40120{s}^{3}+45513{s}^{2}-22308s+7920\right)}{2{\left(1370{s}^{3}-10212{s}^{2}+11745s-6930\right)}^{2}}>0$ and $\frac{\partial {\tilde{Q }}^{O}}{\partial s}=-\frac{108\alpha \left(16598{s}^{4}-40120{s}^{3}+45513{s}^{2}-22308s+7920\right)}{2{\left(1370{s}^{3}-10212{s}^{2}+11745s-6930\right)}^{2}}<0$. (ii) Comparing gross profits, we have that ${\tilde{v }}_{A}-{\tilde{v }}_{B}=-\frac{27s\left(27224{s}^{5}-221750{s}^{4}+477368{s}^{3}-562697{s}^{2}+346412s-108416\right)}{1370{s}^{3}-10212{s}^{2}+11745s-6930}>0$ for $\forall$ *s*.

### B.4 Second-stage equilibrium in the post-merger game

Maximizing (15) with respect to TEUs quantities at either the integrated or non-integrated terminal, i.e., $\partial {\tilde{v }}_{M}\left({\tilde{q }}_{i,M}^{T},{\tilde{q }}_{i,M}^{O};{\tilde{f }}_{M}^{T},{\tilde{f }}_{M}^{O}\right)/\partial {\tilde{q }}_{i,M}^{T}=0$ and $\partial {\tilde{v }}_{M}\left({\tilde{q }}_{i,M}^{T},{\tilde{q }}_{i,M}^{O};{\tilde{f }}_{M}^{T},{\tilde{f }}_{M}^{O}\right)/\partial {\tilde{q }}_{i,M}^{O}=0$, for $i=A,B$, yields second-stage quantities in (16). Analysing the impact of terminal fees, $\frac{\partial {\tilde{q }}_{M}^{T}\left({\tilde{f }}_{M}^{T},{\tilde{f }}_{M}^{O}\right)}{\partial {\tilde{f }}_{M}^{T}}=-\frac{18}{17}\left(1-s\right)<0, \frac{\partial {\tilde{q }}_{M}^{T}\left({\tilde{f }}_{M}^{T},{\tilde{f }}_{M}^{O}\right)}{\partial {\tilde{f }}_{M}^{O}}=\frac{16}{17}>0, \frac{\partial {\tilde{q }}_{M}^{O}\left({\tilde{f }}_{M}^{T},{\tilde{f }}_{M}^{O}\right)}{\partial {\tilde{f }}_{M}^{O}}=-\frac{18}{17}<0, \frac{\partial {\tilde{q }}_{M}^{O}\left({\tilde{f }}_{M}^{T},{\tilde{f }}_{M}^{O}\right)}{\partial {\tilde{f }}_{M}^{T}}=\frac{16}{17}\left(1-s\right)>0$. The vertical integration raise (shrink) the integrated (non integrated) terminal’s supply, i.e., $\frac{\partial {\tilde{q }}_{M}^{T}\left({\tilde{f }}_{M}^{T},{\tilde{f }}_{M}^{O}\right)}{\partial s}=\frac{18}{17}{\tilde{f }}_{M}^{T}>0$ and $\frac{\partial {\tilde{q }}_{M}^{O}\left({\tilde{f }}_{M}^{T},{\tilde{f }}_{M}^{O}\right)}{\partial s}=-\frac{16}{17}{\tilde{f }}_{M}^{T}<0$.

### B.5 First-stage equilibrium in the post-merger game

To derive the first-stage equilibrium, we solve $\frac{\partial {\tilde{v }}_{T}^{M}}{\partial {\tilde{f }}_{M}^{T}}=0$ and $\frac{\partial {\tilde{v }}_{O}^{M}}{\partial {\tilde{f }}_{M}^{O}}=0$ conduces to a two-equation system of best-reply functions ${\tilde{f }}_{M}^{T}\left({\tilde{f }}_{M}^{O}\right)=\frac{\alpha +8{\tilde{f }}_{M}^{O}}{9(2-s)}$ and ${\tilde{f }}_{M}^{O}\left({\tilde{f }}_{M}^{T}\right)=\frac{1}{9}\left(\frac{\alpha }{2}+4{\tilde{f }}_{M}^{T}(1-s)\right)$, whose solution yields equilibrium terminal fees in (17). As regards the impact of shares, $\frac{\partial {\tilde{f }}_{M}^{T}}{\partial s}=\frac{637\alpha }{{\left(130-49s\right)}^{2}}>0$, while $\frac{\partial {\tilde{f }}_{M}^{O}}{\partial s}=-\frac{468\alpha }{{\left(130-49s\right)}^{2}}<0$. Negative (positive) demand changes lower (raise): $\frac{\partial {\tilde{f }}_{M}^{T}}{\partial \alpha }>0$ and $\frac{\partial {\tilde{f }}_{M}^{O}}{\partial \alpha }>0.$

### B.6 Equilibrium outcomes in the post-merger game

For increasing shares, we get $\frac{\partial {\tilde{Q }}_{M}^{T}}{\partial s}=\frac{22932\alpha }{17{\left(130-49s\right)}^{2}}>0$, and $\frac{\partial {\tilde{Q }}_{M}^{O}}{\partial s}=-\frac{16848\alpha }{17{\left(130-49s\right)}^{2}}<0$, while $\frac{\partial {\tilde{v }}_{M}}{\partial s}=\frac{4212{\alpha }^{2}(34s-169)}{17{\left(130-49s\right)}^{3}}>0$. As the demand parameter $\alpha$ is still a multiplicative factor, demand slumps lower equilibrium figures.

### B.7 Proof of Proposition 2

The difference between post- and pre-merger joint gross profits yields $\Delta {\tilde{v }}_{M}\triangleq {\tilde{v }}_{M}-\left({\tilde{v }}_{A}+{\tilde{v }}_{B}\right)=\frac{{\alpha }^{2}\left(60747363848{s}^{8}-977672670496{s}^{7}+5800298902181{s}^{6}-18009144958440{s}^{5}+33882162787242{s}^{4}-40546961704656{s}^{3}+31256784217800{s}^{2}-14439729189600s+3310087809600\right)}{68{\left(67130{s}^{4}-678488{s}^{3}+1903065{s}^{2}-1866420s+900900\right)}^{2}}$ which is a positive function for any *s*. Analysing the sensitivity to demand shocks, we check that $\frac{\partial \stackrel{\sim }{\delta }}{\partial s}<0$, implying that a stronger integration reduces the sensitivity to demand shocks. As for the comparison between throughputs, we prove that ${\tilde{Q }}_{M}^{T}-{\tilde{Q }}_{M}^{O}=\frac{18\alpha s}{\left(130-49s\right)}>0$, so more TEUs are handled at the integrated terminal. Overall, $\Delta {\tilde{Q }}_{M}\triangleq {\tilde{Q }}_{M}-\tilde{Q }=\frac{3\alpha \left(16456{s}^{4}+110015{s}^{3}-396912{s}^{2}+380268s-171600\right)}{17\left(67130{s}^{4}-678488{s}^{3}+1903065{s}^{2}-1866420s+90090\right)}<0$ for any *s*.

## Footnotes

1 Multiple scenarios have been tested (e.g. multiple ownership status, presence of a third independent terminal, mixed service strategies) but results were consistent with the one presented in this paper. For computational simplicity and effectiveness in discussing the results, only the two scenarios above will be discussed. Authors are available for sharing alternative model outcomes.

2 In this paper, we refer to integrated terminals as the ones that are leased out to a company in which at least part of the shares is owned by a shipping company (i.e., a vertically integrated company). Moreover, we use the term “Pure stevedore terminal operators” in the case terminals are either publicly or privately managed by companies without directly or indirectly shareholding by shipping companies.

3 In practice, most partial ownerships in terminals are joint ventures with other terminal operators, many of which are integrated with shipping lines. Several scenarios have been tested and outcomes are consistent with the ones presented in this paper. Other elaborations are available to readers upon request.

4 See details in the Appendix A.1.

5 See details in the Appendix A.3.

6 In this modelling, we analyse merger incentives taking into account demand conditions, captured by $\alpha$. Instead of assuming stochastic changes in the market size, without loss of significance we consider merger projects occurring for a given pre-merger equilibrium demand (see Qiu and Zhou, 2007). Lower (higher) values of $\alpha$ would imply a slump (boom) in the demand of TEUs to be shipped.

7 See Appendix A.4.

8 See details in the Appendix A.5.

9 See details in the Appendix A.6.

10 See details in the Appendix B.1.

11 See details in the Appendix B.2.

12 See details in the Appendix B.3.

13 See details in the Appendix B.4.

14 See details in the Appendix B.5.

15 See details in the Appendix B.6.

16 See details in the Appendix B.7.

## References

[CR1] Achurra-Gonzalez PE, Angeloudis P, Goldbeck N, Zavistas K, Stettler MEJ (2019). Evaluation of port disruption impacts in the global liner shipping network. Journal of Shipping and Trade.

[CR2] Alexandrou G, Gounopoulos D, Thomas H (2014). Mergers and Acquisitions in Shipping. Transportation Research Part E.

[CR3] Alix Y, Slack B, Comtois C (1999). Alliance or Acquisition? Strategies for Growth in the Container Shipping Industry, the Case of CP Ships. Journal of Transport Geography.

[CR4] Álvarez-Sanjaime Ó, Cantos-Sánchez P, Moner-Colonques R, Sempere-Monerris JJ (2013). Vertical Integration and Exclusivities in Maritime Freight Transport. Transportation Research Part E.

[CR5] Baumol W, Ordover J (1994). On the Perils of Vertical Control by a Partial Owner of a Downstream Enterprise. Revue D'economie Industrielle.

[CR6] Brito, D., L.M.B. Cabral, and H. Vasconcelos. 2016. Competitive Effects of Partial Control in an Input Supplier, CEPR Discussion Paper No. DP11397, Available at SSRN: https://ssrn.com/abstract=2814072

[CR7] Chen CC, Tsai YH, Schonfeld P (2016). Schedule Coordination, Delay Propagation, and Disruption Resilience in Intermodal Logistics Networks. Journal of Transportation Research Board..

[CR8] Chen Y (2001). On Vertical Mergers and Their Competitive Effects. Rand Journal of Economics.

[CR9] Crotti D, Ferrari C, Tei A (2020). Merger Waves and Alliance Stability in container Shipping. Maritime Economics & Logistics.

[CR10] Das S (2011). To Partner or to Acquire? A Longitudinal Study of Alliances in the Shipping Industry. Maritime Policy and Management.

[CR12] Ferrari C., and A. Tei. 2021. Is The Shipping Industry Encountering a Black Swan Event?, *Proceedings of World of shipping conference 2021*, Lisbon, Portugal, ISBN: 978-989-33-1436-4.

[CR13] Ferrari C, Parola F, Benacchio M (2008). Network Economies in Liner Shipping: The Role of Home Markets. Maritime Policy and Management.

[CR14] Ferrari C, Tei A (2020). Effects of BRI Strategy on Mediterranean Shipping Transport. Journal of Shipping and Trade.

[CR15] Fronmueller, M.P., and R. Reed. 1996. The competitive advantage potential of vertical integration. *Omega*, 24 (6): 715–726. https://EconPapers.repec.org/RePEc:eee:jomega:v:24:y:1996:i:6:p:715-726

[CR16] Greenlee P, Raskovich A (2006). Partial Vertical Ownership. European Economic Review.

[CR17] Hamilton J, Mqasqas I (1996). Double Marginalization and Vertical Integration: New Lessons from Extensions of the Classic Case. Southern Economic Journal.

[CR18] Haralambides HE, Cariou P, Benacchio M (2002). Costs, Benefits and Pricing of Dedicated Container Terminals. International Journal of Maritime Economics.

[CR19] Hoffmann, J. and J. Hoffmann. 2021. *Bigger Ships and Fewer Companies—Two Sides of the Same Coin*. Accessible at: https://unctad.org/news/bigger-ships-and-fewer-companies-two-sides-same-coin.

[CR20] IMF (2021), World Economic Outlook 2021, April 2021.

[CR21] ITF - International Transport Forum (2018). The Impact of Alliances in Container Shipping.

[CR22] ITF - International Transport Forum (2019). Container Shipping in Europe.

[CR23] Kaselimi E, Notteboom T, De Borger B (2011). A Game Theoretical Approach to Competition between Multi-User Terminals: The Impact of Dedicated Terminals. Maritime Policy & Management.

[CR24] LaPorta R, de Silanes FL, Shleifer A, Vishny R (2000). Investor Protection and Corporate Governance. Journal of Financial Economics.

[CR25] Loertscher S, Reisinger M (2014). Market structure and the competitive effects of vertical integration. RAND Journal of Economics.

[CR26] Mathewson GF, Winter RA (1984). An Economic Theory of Vertical Restraints. The RAND Journal of Economics.

[CR27] McAfee, R.P., M.A. Williams. 1992. Horizontal mergers and Antitrust Policy, *The Journal of Industrial Economics* 40 (2): 181–187. http://www.jstor.org/stable/2950509.

[CR28] Meersman, H., C. Sys, E. van de Voorde, and T. Vanelslander. 2015. *Competition Issues in Liner Shipping*. OECD, https://www.oecd.org/officialdocuments/publicdisplaydocumentpdf/?cote=DAF/COMP/WP2(2015)5&docLanguage=En.

[CR29] Merck O (2020). Quantifying Tax Subsidies to Shipping. Maritime Economics & Logistics.

[CR30] Midoro R, Musso E, Parola F (2005). Maritime Liner Shipping and the Stevedoring Industry: Market Structure and Competition Strategies. Maritime Policy & Management.

[CR31] Midoro R, Pitto A (2000). A Critical Evaluation of Strategic Alliances in Liner Shipping. Maritime Policy and Management.

[CR32] Motta M (2004). Competition Policy: Theory and Practice.

[CR33] Motta M, Vasconcelos H (2005). Efficiency Gains and Myopic Antitrust Authority in a Dynamic Merger Game. International Journal of Industrial Organization.

[CR34] Notteboom T., A. Pallis, and J.P. Rodrigue. 2020. Disruptions and resilience in global container shipping and ports: the COVID-19 pandemic versus the 2008–2009 financial crisis, *Maritime Economics & Logistics*, in press.

[CR35] Notteboom T (2002). Consolidation and Contestability in the European Container Handling Industry. Maritime Policy & Management.

[CR36] Notteboom T, Haralambides H (2020). Port Management and Governance in a Post-COVID-19 Era: Quo Vadis?. Maritime Economics & Logistics.

[CR37] Notteboom T, Rodrigue JP (2012). The Corporate Geography of Global Container Terminal Operators. Maritime Policy & Management.

[CR38] O'Brien DP, Salop SC (2000). Competitive Effects of Partial Ownership: Financial Interest and Corporate Control. Antitrust Law Journal.

[CR39] OECD. 2015. *Competition Issues in Liner Shipping*. Accessible at: https://www.oecd.org/daf/competition/competition-issues-in-liner-shipping.htm

[CR40] Perry M, Porter R (1985). Oligopoly and the Incentive for Horizontal Merger. American Economic Review.

[CR41] Qiu LD, Zhou W (2007). Merger Waves: A Model of Endogenous Mergers. RAND Journal of Economics.

[CR42] Rahman M, Kim J, Laratte B (2020). Disruption in Circularity? Impact Analysis of COVID-19 on Ship Recycling Using Weibull Tonnage Estimation and Scenario Analysis Method. Resources, Conservation and Recycling.

[CR43] Rey P, Tirole J, Armstrong M, Porter R (2007). A Primer on Foreclosure. Handbook of Industrial Organization.

[CR44] Riordan MH (1991). Ownership Without Control: Toward a Theory of Backward Integration. Journal of the Japanese and International Economies.

[CR45] Riordan MH (1998). Anticompetitive Vertical Integration by a Dominant Firm. American Economic Review.

[CR46] Riordan MH, Buccirossi P (2008). Competitive Effects of Vertical Integration. Handbook of Antitrust Economics.

[CR47] Rodrigue JP, Notteboom T (2009). The Terminalization of Supply Chains: Reassessing the Role of Terminals in Port/Hinterland Logistical Relationships. Maritime Policy & Management.

[CR48] Slack B, Fremont A (2005). Transformation of Port Terminal Operations from the Local to the Global. Transport Reviews.

[CR49] Slade M, Kwoka JE (2020). Second Thoughts on Double Marginalization. Antitrust.

[CR50] Spengler JJ (1950). Vertical Integration and Antitrust Policy. Journal of Political Economy.

[CR51] TradeWinds. 2021. www.tradewindsnews.com

[CR52] UNCTAD (2020). Review of Maritime Transport.

[CR53] Vasconcelos H (2010). Efficiency Gains and Structural Remedies in Merger Control. Journal of Industrial Economics.

[CR54] van de Voorde, E. and T. Vanelslander, 2009. *Market Power and Vertical and Horizontal Integration in the Maritime Shipping and Port Industry*. OECD/ITF Joint Transport Research Centre Discussion Papers, No. 2009/02, OECD Publishing, Paris, 10.1787/227458312782.

[CR55] Wang K, Ng A, Lam J, Fu X (2012). Cooperation or Competition? Factors and Conditions Affecting Regional Port Governance in South China. Maritime Economics & Logistics.

[CR56] Wilmsmeier G, Sánchez RJ (2015). Evolution of Shipping Networks—Current Challenges in Emerging Markets. Zeitschrift Für Wirtschaftsgeographie.

[CR57] Yip TL, Liu JJ, Fu X, Feng J (2014). Modeling the Effects of Competition on Seaport Terminal Awarding. Transport Policy.

[CR58] Zheng S, Luo M (2021). Competition or Cooperation? Ports’ Strategies and Welfare Analysis Facing Shipping Alliances. Transportation Research Part E.

[CR59] Zhu S, Zheng S, Ge YE, Fu X, Sampaio B, Jang C (2019). Vertical Integration and its Implications to Port Expansion. Maritime Policy & Management.

